# Supplementary material for: Conditional chemoconnectomics (cCCTomics) as a strategy for efficient and conditional targeting of chemical transmission
Source: eLife. 2024 Apr 30;12:RP91927. doi: 10.7554/eLife.91927 (PMC11060718; doi:10.7554/eLife.91927)
Supplement: Figure 3—source data 3. [file elife-91927-fig3-data3.zip › Figure 3 source data3 part 2.zip!/Figure 3-Source data 3-part2/1063-1-CRISPResso_Report_az97gqtx.zip!/CRISPResso_on_1063_3/1c.Alignment_barplot.pdf]

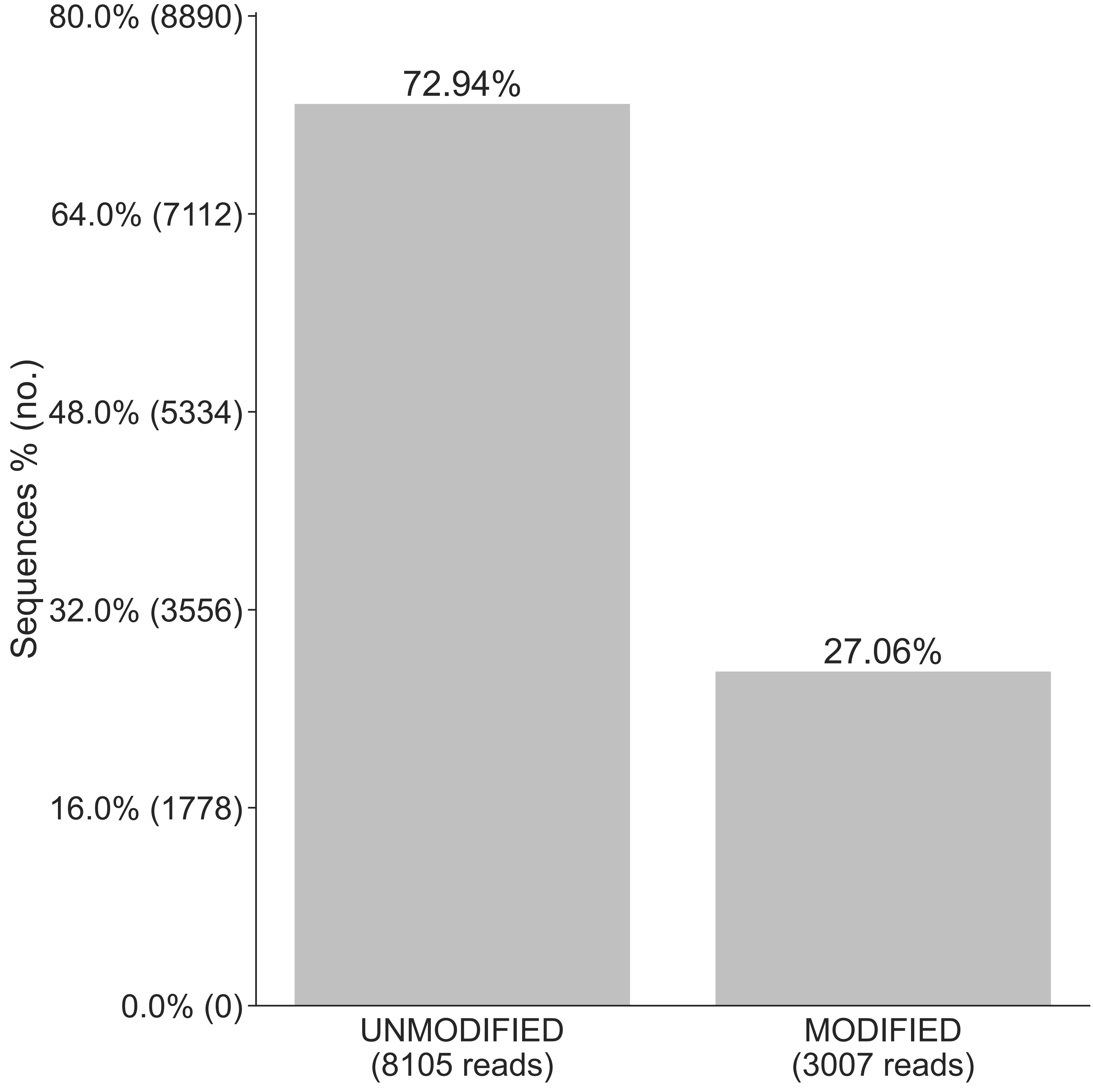

80.0% (8890)
64.0% (7112)
48.0% (5334)
32.0% (3556)
16.0% (1778)
0.0% (0)
Sequences % (no.)
72.94%
27.06%
UNMODIFIED
(8105 reads)
MODIFIED
(3007 reads)
